# Lithology Controls on Arbuscular Mycorrhizal Fungi Across Bulk Soil and Rock–Soil Interface

**DOI:** 10.3390/microorganisms14051023

**Published:** 2026-04-30

**Authors:** Rui Pan, Hao Hu, Kaixun Yang, Dan Xiao, Cong Wang, Hanqing Wu, Qiumei Ling, Mingming Sun, Wei Zhang, Kelin Wang

**Affiliations:** 1 Guangxi Key Laboratory of Forest Ecology and Conservation, State Key Laboratory for Conservation and Utilization of Subtropical Agro-Bioresources, School of Forestry, Guangxi University, Nanning 530004, China; pan__r@163.com (R.P.); hujiaahao@163.com (H.H.); yangkaixun0226@163.com (K.Y.); 2 Institute of Subtropical Agriculture, Chinese Academy of Sciences, Changsha 410125, China; hqwu@isa.ac.cn (H.W.); lingqiumei22@mails.ucas.ac.cn (Q.L.); smm23@mails.ucas.ac.cn (M.S.); zhangw@isa.ac.cn (W.Z.); kelin@isa.ac.cn (K.W.); 3 Huanjiang Observation and Research Station for Karst Ecosystem, Guangxi Key Laboratory of Karst Ecological Processes and Services, Institute of Subtropical Agriculture, Chinese Academy of Sciences, Huanjiang 547100, China; 4 University of Chinese Academy of Sciences, Beijing 100039, China

**Keywords:** AMF community, dolomite, limestone, clastic rock, rock–soil interface

## Abstract

Arbuscular mycorrhizal fungi (AMF) are vital for nutrient cycling, but how lithology across bulk soil and the rock–soil interface influence AMF communities remains poorly understood. We investigated the effects of karst (dolomite, limestone) and non-karst (clastic rock) lithologies across bulk soil and the rock–soil interface on AMF diversity, community composition, and co-occurrence networks in southwest China. AMF diversity did not differ among lithologies or between bulk soil and rock–soil interface, whereas community composition showed significant differences across lithology. The relative abundance of *Glomus* was lower in karst than in non-karst, whereas *Paraglomus* showed the opposite pattern. Co-occurrence network analysis revealed that karst soils exhibited higher numbers of nodes and edges but lower network density, transitivity, betweenness centrality, and average path length compared to non-karst soils. Within the same dolomite and limestone, network properties were similar between the rock–soil interface and bulk soil. Soil pH, exchangeable Ca^2+^ and Mg^2+^, total nitrogen, and nitrate nitrogen were negatively correlated with *Glomus* and network properties (e.g., number of nodes and edges), while ammonium nitrogen showed positive correlations. Our results indicate that lithology exerts a stronger influence than soil compartment on AMF community composition and interspecific interactions, emphasizing the key role of lithological substrates in regulating AMF communities.

## 1. Introduction

Arbuscular mycorrhizal fungi (AMF) are symbiotic fungi that colonize plant roots and form specialized structures, such as arbuscules, hyphae, and vesicles, to establish mutualistic associations [1]. This symbiosis is remarkably widespread, occurring in approximately 80% of terrestrial plant species, including many ecologically and economically important trees [2,3]. AMF substantially enhance plant nutrient acquisition, particularly phosphorus (P), and increase plant tolerance to a wide range of environmental stresses [4]. In degraded ecosystems, where soil nutrient availability is limited and vegetation recovery is slow, AMF facilitate plant establishment and succession by improving nutrient uptake, thereby promoting higher plant diversity [5,6]. By supplying essential resources to their host plants, AMF play a critical role in sustaining ecosystem functioning and resilience [7,8].

Lithology exerts a strong influence on AMF communities by regulating soil geochemistry. Karst landscapes are widely distributed in southwestern China and are predominantly developed on carbonate rocks, such as dolomite and limestone [9]. In contrast to non-karst soils derived from clastic rocks, karst soils formed on carbonate substrates exhibit distinct soil physicochemical properties and vegetation characteristics [10,11], which in turn shape AMF diversity and community structure [12,13,14]. In karst ecosystems, shallow soil layers commonly constrain plant growth and species richness, often resulting in lower AMF abundance. Moreover, elevated concentrations of Ca^2+^ in carbonate-derived soils promote the formation of insoluble Ca–P compounds, thereby reducing P availability and potentially limiting the efficiency of AMF symbiosis with host plants [15,16]. Lithological variation also modifies soil environmental conditions, including moisture regime and soil texture, which may restrict AMF hyphal proliferation and nutrient transfer to host plants [13,14]. Although previous studies have documented differences in AMF communities between karst and non-karst soils, the role of the rock–soil interface across contrasting lithologies in structuring AMF communities remains poorly understood.

The rock–soil interface, located adjacent to weathering rock surfaces, is characterized by higher mineral reactivity and greater nutrient availability than the surrounding bulk soil. Weathering processes at the rock–soil interface release soluble nutrients, such as available nitrogen (N) and P, potentially alleviating nutrient limitations for AMF [17,18,19]. In addition, microfractures and mineral surfaces at the rock–soil interface may serve as preferential sites for AMF hyphal attachment and expansion, thereby enhancing symbiotic interactions with host plants [20]. By contrast, in bulk soil, intense microbial competition for N and P may force AMF to rely more heavily on carbon inputs from their host plants and to adjust their metabolic strategies accordingly [21]. Despite increasing recognition of lithology as a key driver of AMF communities in karst ecosystems, several knowledge gaps remain. First, previous studies have primarily focused on comparing dolomite and limestone, while systematic comparisons between dolomite, limestone, and clastic rock soils are scarce. Second, few studies have directly compared AMF diversity, community composition, and interactions between the rock–soil interface and bulk soil across contrasting lithologies.

To address these gaps, this study simultaneously examined AMF communities across three lithologies (dolomite, limestone, and clastic rock) and two soil compartments (bulk soil and rock–soil interface), with the aim of elucidating how lithology and soil microhabitat jointly shape AMF diversity, community composition, and interspecific interactions in subtropical forests of southwest China.

## 2. Materials and Methods

### 2.1. Site Description

The study was conducted in Huanjiang County, Guangxi Zhuang Autonomous Region, southeastern China (24°43′–25°00′ N, 107°51′–108°43′ E). The region has a typical subtropical monsoon humid climate, with a mean annual temperature (MAT) of 20.3 °C and mean annual precipitation (MAP) of 1399.7 mm. The climate is characterized by a warm and wet season from April to October and a cold and dry season from November to March.

### 2.2. Sample Collection and Analysis

**Figure 1:**
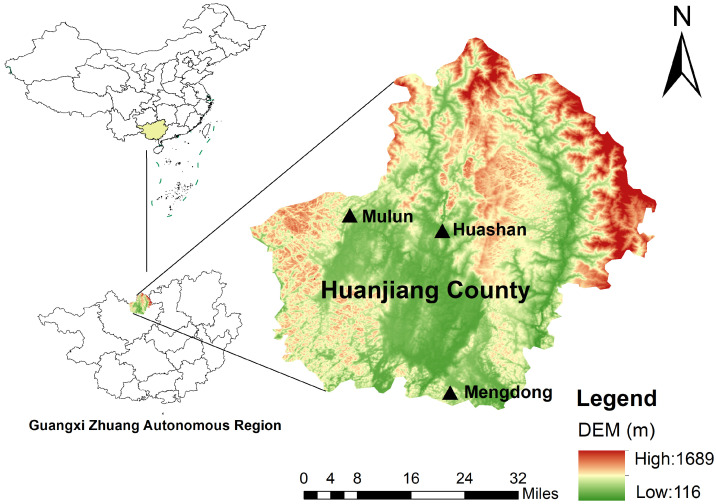
Location of the study area and distribution of the sampling sites.

Soil samples were collected from September to October 2022. We selected karst forests in Mengdong (dolomite) and Mulun (limestone), along with non-karst forests in Huashan (clastic rocks), as study sites (Figure 1). Karst areas were predominantly composed of dolomite and limestone, whereas non-karst areas were dominated by clastic rocks [22]. The soils in the dolomite and limestone plots are calcareous soils developed on carbonate bedrock and can be generally classified as Calcaric Cambisols according to the World Reference Base for Soil Resources, whereas soils in clastic rock plots are Haplic Acrisols [23]. For each lithology, six 20 × 20 m forest plots were established, based on geochemical consistency, lithology type, and similar land-use histories as recorded by local forestry authorities. Accordingly, dolomite bulk soil (DS), dolomite rock–soil interface (DI), limestone bulk soil (LS), and limestone rock–soil interface (LI) were designated as karst representatives, while clastic rock bulk soil (CS) represented non-karst sites [24].

Soil samples were collected from the top 0–15 cm layer within each 20 × 20 m plot, with a minimum distance of 60 m between adjacent plots. In each plot, a 5 cm diameter steel corer was used to extract 20 subsamples, which were then thoroughly mixed to represent the average conditions of the plot. During the collection of soil from the rock–soil interface, undisturbed soil adjacent to the rock surface (within 2 cm from the contact boundary) was carefully sampled. All samples were transported to the laboratory and divided into two portions. The fresh soil was sieved through a 2 mm mesh to remove coarse debris prior to further analysis. A portion of the soil was stored at −80 °C for subsequent DNA extraction, while the remaining soil was either stored at 4 °C or air-dried for the analysis of physical and chemical properties.

### 2.3. Soil Physicochemical Properties

Soil physicochemical properties were determined using established methods [25,26]. Soil pH was measured with a pH meter (FE20K; Mettler-Toledo, Zurich, Switzerland) at a soil-to-water ratio of 1:2.5. Soil texture (clay + silt) was analyzed using a laser particle size analyzer (Mastersizer 2000; Malvern Instruments, Malvern, UK) [27]. Soil organic carbon (SOC) and soil total nitrogen (STN) were quantified using an elemental analyzer (Vario EL III; Elementar GmbH, Langenfeld, Germany) [28]. Fresh soil subsamples were extracted with 2 M KCl (10 g soil: 50 mL KCl), filtered through pre-combusted glass fiber filters, and analyzed for total dissolved nitrogen (TDN), ammonium (NH_4_^+^), and nitrate (NO_3_^−^) using an autoanalyzer (FIAstar 5000; FOSS Tecator, Höganäs, Sweden) [26]. Total organic nitrogen (TON) was calculated as STN minus NH_4_^+^ and NO_3_^−^, whereas dissolved organic nitrogen (DON) was calculated as TDN minus NH_4_^+^ and NO_3_^−^. Microbial biomass carbon (MBC) was determined using the chloroform fumigation–extraction method [29,30]. Reactive soil mineral composition, including exchangeable Ca^2+^ and Mg^2+^ ((Ca + Mg)_exe_), poorly crystalline oxyhydroxides of iron and aluminum ((Fe + Al)_o_), and pedogenic iron and aluminum ((Fe + Al)_d_), was analyzed. Mineral composition of rocks (Ca, Al, Mg, Fe), as well as reactive soil mineral composition and mineral protection indices ((Ca + Mg)_exe_ and (Fe + Al)_o_), were determined using inductively coupled plasma optical emission spectrometry (ICP-OES; Agilent 5100, Agilent Technologies, Santa Clara, CA, USA) [31]. In this study, soil physicochemical properties were derived from our previously published study [32].

### 2.4. Soil DNA Extraction and PCR Amplification

Total soil DNA was extracted from 0.5 g of freeze-dried soil using the FastDNA SPIN Kit (MP Biomedicals, Santa Ana, CA, USA) according to the manufacturer’s instructions. DNA integrity was evaluated by 1% (*w*/*v*) agarose gel electrophoresis under constant voltage, and DNA concentration and purity were determined using a NanoDrop spectrophotometer (NanoDrop Technologies, Wilmington, DE, USA). After confirming that the DNA met the quality requirements for subsequent analyses, the extracts were aliquoted and stored at −20 °C until PCR amplification.

For high-throughput sequencing, amplicons were generated using a nested PCR approach. The primer pairs AML1 (5′-ATCAACTTCGATGGTAGGATAGA-3′) and AML2 (5′-GAACCCAAACACTTTGGTTCC-3′) were used in the first-round PCR, while AMV4.5NF (5′-AAGCTCGTAGTTGAATTTCG-3′) and AMDGR (5′-CCCAACTATCCCTAATTAATCAT-3′) were used in the second-round PCR. A unique 6 bp barcode was attached to the AMV4.5NF/AMDGR primer set to distinguish individual samples. The first PCR was performed in a 20 μL reaction mixture containing 1 μL of template DNA, 10 μL of 2× SYBR Premix Ex Taq (Takara, Kusatsu, Shiga, Japan), 0.5 μL of each primer, and 8 μL of nuclease-free water. The resulting PCR products were diluted 50-fold and used as templates for the second PCR. The first PCR cycling conditions were as follows: an initial denaturation at 94 °C for 5 min, followed by 35 cycles of denaturation at 94 °C for 30 s, annealing at 58 °C for 45 s, and extension at 72 °C for 60 s, with a final extension at 72 °C for 10 min. The second PCR was performed with an initial denaturation at 94 °C for 3 min, followed by 30 cycles of 94 °C for 45 s, 60 °C for 45 s, and 72 °C for 60 s, and a final extension at 72 °C for 10 min. PCR products were purified prior to sequencing, and the purified amplicons were sequenced on an Illumina HiSeq 2500 platform (Illumina, San Diego, CA, USA) [33,34].

### 2.5. Sequence Analysis

All AMF sequences were processed using QIIME 2 (version 2020.2). In this study, sequences were clustered into operational taxonomic units (OTUs) at 97% sequence similarity using the q2-vsearch plugin in QIIME2. Taxonomic assignment was conducted using the q2-feature-classifier plugin trained on the MaarjAM database. Taxonomic ranks were cross-validated against the NCBI taxonomy database using an in-house R script. Alpha-diversity indices were calculated using the RAM package and visualized with ggplot2 in R version 4.5.0 (R Foundation for Statistical Computing, Vienna, Austria) [35,36].

### 2.6. Statistical Analysis

All data were tested for normality and homogeneity of variance prior to analysis. The effects of lithology between bulk soil and the rock–soil interface on soil physicochemical properties, microbial abundance, and diversity indices were assessed using one-way analysis of variance (ANOVA), followed by the least significant difference (LSD) test for post hoc comparisons (*p* < 0.05) in SPSS 26.0 (SPSS Inc., Chicago, IL, USA). Non-metric multidimensional scaling (NMDS) based on OTU data was performed using the metaMDS function in the vegan package (v2.6-4, University of Oulu, Oulu, Finland) to visualize variations in AMF community composition across different lithologies and between bulk soil and the rock–soil interface. Pearson correlation analysis was applied to examine relationships between AMF index and soil physicochemical properties. An overall AMF co-occurrence network was constructed using all samples across treatments based on Spearman’s rank correlations among OTUs, retaining statistically significant (*p* < 0.01) associations. Subsequently, sample-specific subnetworks were extracted from the overall network to calculate topological properties for each sample, such as the number of node and degree, density, average path length, and transitivity. Network visualization was performed using the igraph package (v1.2.6, CIRCB, Rome, Italy) with the “sphere” layout. All statistical analyses and graphical visualizations were conducted using SPSS 26.0, Origin 2021 (OriginLab, Hampton, NY, USA), and R version 4.5.0 (R Foundation for Statistical Computing, Vienna, Austria).

## 3. Results

### 3.1. Soil Physicochemical Properties Under Lithology Between Bulk Soil and Rock–Soil Interface

We observed significant variation in soil physicochemical properties across lithology between bulk soil and the rock–soil interface. In bulk soils, karst dolomite and limestone exhibited significantly higher soil pH, soil water content (SWC), soil organic carbon (SOC), soil total nitrogen (STN), nitrate (NO_3_^−^), exchangeable Ca^2+^ and Mg^2+^, microbial biomass carbon (MBC), microbial biomass nitrogen (MBN), and fine particle content (clay + silt) than non-karst clastic rock soils (Figure S1). In contrast, ammonium (NH_4_^+^) concentrations were significantly higher in non-karst clastic rock soils. Within the same karst lithology (dolomite or limestone), we further found that the rock–soil interface consistently exhibited significantly higher soil pH, STN, NH_4_^+^, exchangeable Ca^2+^ and Mg^2+^, and MBC than the corresponding bulk soil (Figure S1).

### 3.2. AMF Diversity and Community Composition Under Lithology Between Bulk Soil and Rock–Soil Interface

**Figure 2:**
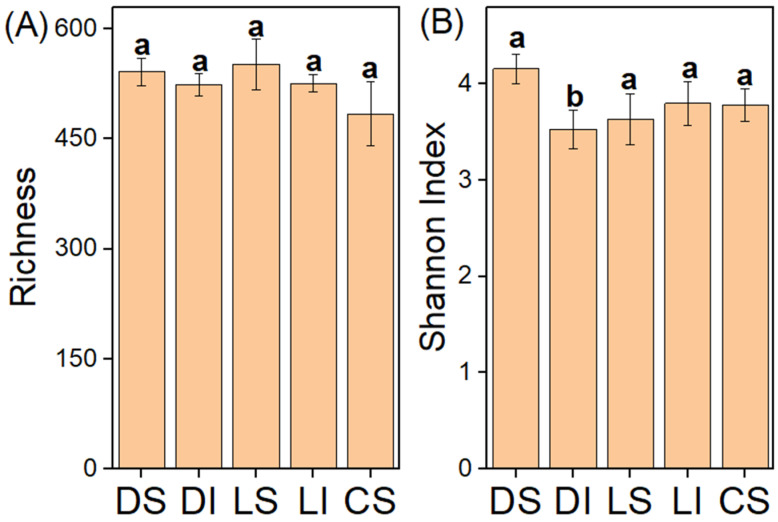
The effect of lithology between bulk soil and the rock–soil interface on AMF diversity. DS, dolomite bulk soil; DI, dolomite rock–soil interface; LS, limestone bulk soil; LI, limestone rock–soil interface; CS, clastic rock bulk soil. Different lowercase letters indicate significant differences among treatments (*p* < 0.05).

**Figure 3:**
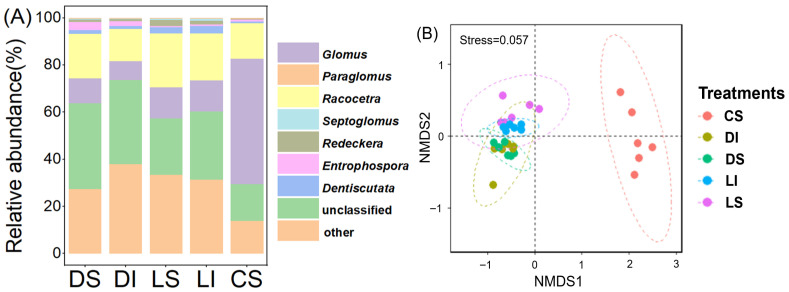

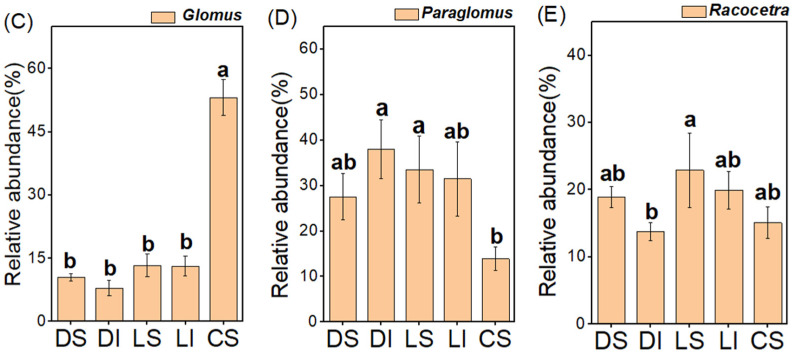
Effects of lithology between bulk soil and the rock–soil interface on AMF community composition, showing NMDS ordination, genus-level relative abundance, and comparisons of the three dominant genera. (**A**) Horizontal stacked bar plot showing the genus-level relative abundance of AMF across different treatments; (**B**) NMDS ordination of AMF community composition based on OTU data, dotted circles represent 95% confidence intervals for each treatment group. Less overlap and greater distance between circles indicate larger differences in community composition. (**C**–**E**) Comparisons of relative abundances of the three dominant genera. DS, dolomite bulk soil; DI, dolomite rock–soil interface; LS, limestone bulk soil; LI, limestone rock–soil interface; CS, clastic rock bulk soil. Different lowercase letters indicate significant differences among treatments (*p* < 0.05).

We found no significant differences in AMF diversity, as indicated by richness and the Shannon index (Figure 2), across lithologies or between bulk soil and the rock–soil interface. In contrast, AMF community composition differed significantly between karst and non-karst soils (Figure 3).

Across all treatments, *Glomus*, *Paraglomus*, and *Racocetra* were the dominant genera. Specifically, the relative abundance of *Glomus* was significantly lower in karst dolomite soils (10.5%) and limestone soils (13.3%) than in non-karst soils (53.2%), whereas *Paraglomus* showed the opposite pattern (dolomite: 27.5%; limestone: 33.5%; non-karst: 13.9%) (Figure 3C–E). Under bulk soil conditions, the relative abundance of *Glomus* was significantly higher in non-karst clastic rock soils than in karst dolomite and limestone soils (Figure 3C), whereas *Paraglomus* was significantly more abundant in karst dolomite and limestone soils (Figure 3D). No significant differences in the relative abundance of *Racocetra* were detected among lithologies (Figure 3E). Within the same karst lithology (dolomite or limestone), we observed no significant differences in the relative abundances of these three dominant genera between bulk soil and the rock–soil interface (Figure 3).

### 3.3. Co-Occurrence Networks of AMF Taxa

**Figure 4:**
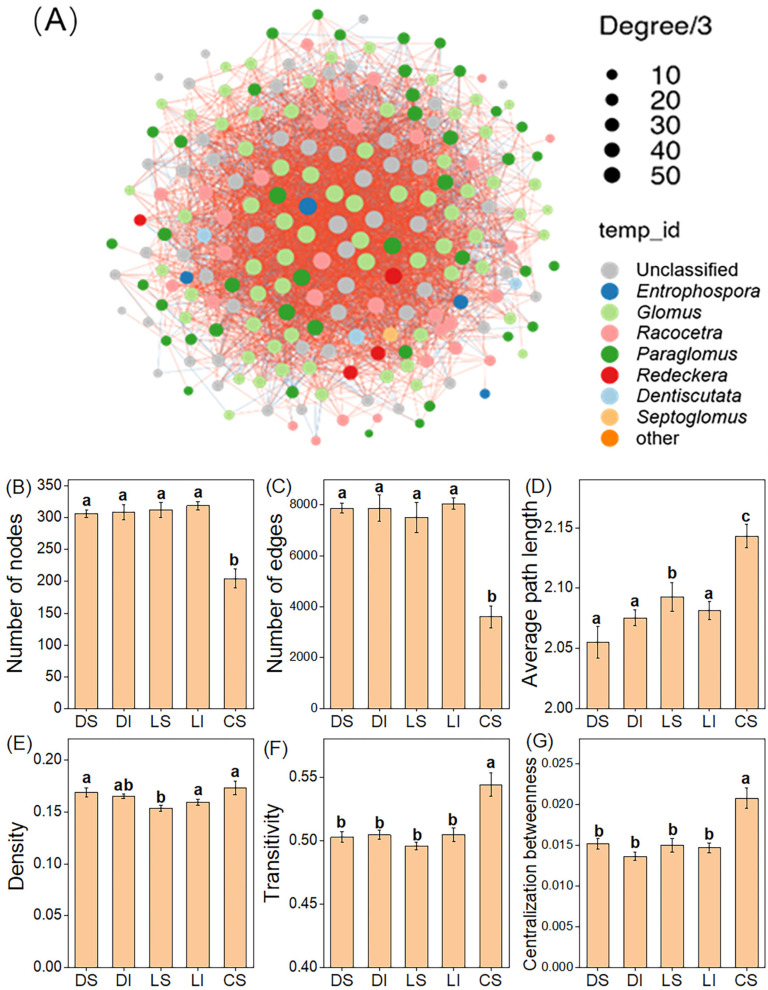
Co-occurrence networks and topological properties of AMF taxa in bulk soil and at the rock–soil interface across karst and non-karst forest ecosystems. (**A**) Co-occurrence networks based on OTUs across all samples. Each point represents an OTU, colored by genus. (**B**–**G**) Topological properties of AMF taxa. DS, dolomite bulk soil; DI, dolomite rock–soil interface; LS, limestone bulk soil; LI, limestone rock–soil interface; CS, clastic rock bulk soil. Different lowercase letters indicate significant differences among treatments (*p* < 0.05).

We constructed AMF co-occurrence networks at the OTU level and observed pronounced differences in network topology among lithologies but not between bulk soil and the rock–soil interface (Figure 4). Under comparable bulk soil conditions, non-karst clastic rock soils compared to karst dolomite and limestone soils exhibited higher network density (except for dolomite), average path length, transitivity, and centralization betweenness, though there were higher numbers of nodes and edges in karst soils (Figure 4). Within karst systems, we further found that most topological properties were similar in bulk soil than in the rock–soil interface for both dolomite and limestone (Figure 4A,B,E,F); only network density and average path length in the limestone is higher at the rock–soil interface than in the bulk soil (Figure 4C,D).

Across both karst and non-karst forest soils, AMF co-occurrence networks were dominated by *Glomus*, *Paraglomus*, and *Racocetra*. These taxa showed high connectivity and acted as central nodes, forming extensive associations with other genera, including *Dentiscutata*, *Septoglomus*, *Entrophospora*, and *Redeckera*, as well as multiple unclassified taxa (Figure 4A).

### 3.4. Relationships Between AMF Profiles and Soil Properties

**Figure 5:**
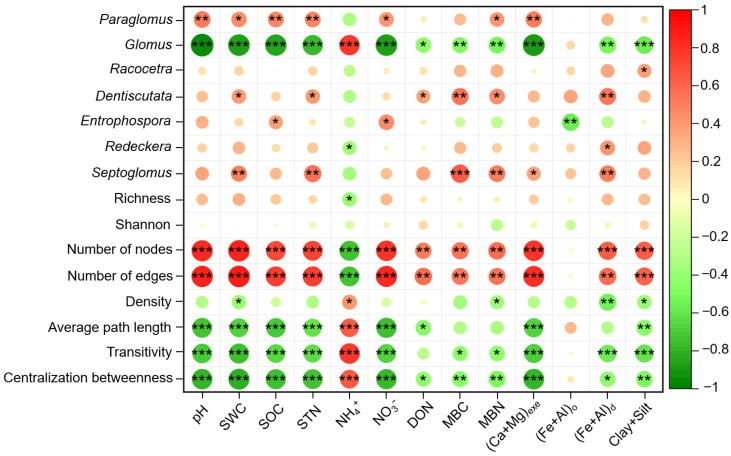
Pearson correlation between the AMF community and soil properties. Color intensity represents the strength of the correlation. Asterisks (*, **, ***) indicate significance levels at *p* < 0.05, *p* < 0.01, and *p* < 0.001, respectively.

We used Pearson correlation analysis to examine relationships between soil physicochemical properties and AMF taxa. Soil pH, SOC, STN, and exchangeable Ca^2+^ and Mg^2+^ showed no significant correlations with AMF richness or Shannon diversity. However, these variables were significantly associated with the relative abundances of the dominant genera (*Paraglomus* and *Glomus*) and with the structural properties of the AMF co-occurrence networks. Specifically, soil pH, SOC, STN, and exchangeable Ca^2+^ and Mg^2+^ were significantly positively correlated with *Paraglomus*, but negatively correlated with *Glomus*. These soil properties were significantly positively correlated with the node and edge numbers of the AMF co-occurrence networks, but negatively correlated with average path length, transitivity, and betweenness centralization. In addition, NO_3_^−^ exhibited strong positive correlations with *Paraglomus* and with network node and edge numbers, but a significant negative correlation with *Glomus*. In contrast, NH_4_^+^ was significantly positively correlated with *Glomus*, but negatively correlated with node and edge numbers of the AMF co-occurrence networks (Figure 5).

**Figure 6:**
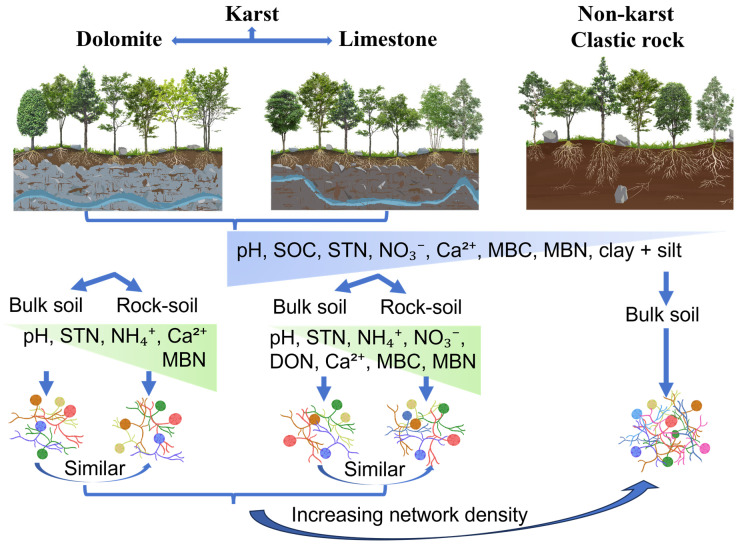
Conceptual model illustrating the relationships among different lithologies, bulk soil, the rock–soil interface, and arbuscular mycorrhizal fungi (AMF) taxa in both karst and non-karst forests. DS, dolomite bulk soil; DI, dolomite rock–soil interface; LS, limestone bulk soil; LI, limestone rock–soil interface; CS, clastic rock bulk soil.

## 4. Discussion

### 4.1. The Effect of Lithology Between Bulk Soil and Rock–Soil Interface on AMF Diversity

This study found that karst dolomite and limestone exhibited higher soil physicochemical properties than non-karst clastic rocks under comparable bulk soil conditions. Carbonate rocks mainly derived from dolomite [CaMg(CO_3_)_2_] contain higher concentrations of CaO and MgO than clastic rocks as reported in previous studies [16,37]. These lithology-controlled geochemical backgrounds promote the dissolution of Ca and Mg and enhance the release of essential elements into the soil. As a result, karst forest soils exhibit higher soil pH, increased exchangeable Ca^2+^ and Mg^2+^, and greater nutrient availability particularly N compared to non-karst clastic rocks [12,38]. Importantly, within both dolomite and limestone systems, the rock–soil interface exhibited higher microbial biomass and greater soil N availability than the surrounding bulk soil. This indicates that the rock–soil interface functions as a nutrient-enriched microhabitat where localized mineral soil interactions and organic matter accumulation enhance microbial activity.

Contrary to our expectations, although soil pH, exchangeable Ca^2+^ and Mg^2+^, and N availability were significantly higher in karst dolomite and limestone than in non-karst clastic rocks and further elevated at the rock–soil interface compared with bulk soil, these differences did not lead to significant changes in AMF diversity. This pattern likely reflects the broad ecological tolerance of many AMF taxa, which allows them to maintain stable richness across contrasting soil physicochemical conditions in both karst and non-karst ecosystems [39,40,41]. In addition, AMF diversity is largely influenced by host plant community and the structure of root-associated niches [42,43]. Previous studies have shown that higher plant diversity can promote greater AMF diversity [44,45]. Although karst ecosystems are often reported to have lower vegetation diversity than non-karst areas [46,47,48], the effects of lithology on AMF diversity may be weakened by the co-occurrence of high pH and elevated exchangeable Ca^2+^, thereby buffering potential differences in AMF diversity across lithologies. Moreover, karst landscapes are characterized by high habitat heterogeneity, which may further buffer or override the effects of soil chemical gradients on AMF diversity. Together, these factors likely contribute to the absence of significant differences in AMF diversity across lithologies. Furthermore, while the rock–soil interface provides a nutrient-rich microhabitat, its limited spatial coverage compared with bulk soil may constrain its impact on overall AMF richness and diversity [49].

### 4.2. Effects of Lithology Between Bulk Soil and Rock–Soil Interface on AMF Community Composition

This study demonstrated that AMF community composition is jointly shaped by lithology between bulk soil and the rock–soil interface, with these factors playing key roles in structuring AMF communities through their effects on soil physicochemical properties [32]. This observation is consistent with findings reported in previous studies. Numerous studies have reported that *Glomus* is the genus with the highest relative abundance among AMF, largely attributed to its strong adaptive capacity to thrive under diverse environmental conditions [39,41,45]. The higher relative abundance of *Glomus* in non-karst clastic rock soils compared with karst dolomite and limestone can be attributed to contrasting soil chemical conditions among lithologies [14,50]. Specifically, *Glomus* exhibited a significant positive relationship with NH_4_^+^, which was more abundant in non-karst clastic rock soils. This pattern is consistent with previous findings that *Glomus* preferentially utilizes NH_4_^+^ and tends to perform better under relatively low total nitrogen availability [51,52,53]. Moreover, the high-pH environment in karst regions has been reported to suppress the growth of *Glomus* [54], while the high exchangeable Ca^2+^ conditions may also inhibit functional expression in some *Glomus* strains [50]. In the present study, significant negative correlations were observed between pH, exchangeable Ca^2+^, and *Glomus* abundance, which further explains the higher relative abundance of *Glomus* in non-karst clastic rock environments. Our finding that *Glomus* is suppressed in karst soils contrasts with several studies from other karst regions, where *Glomus* remained the dominant genus even under high Ca^2+^ conditions [13]. This discrepancy may reflect strain-specific adaptations or differences in soil Ca^2+^ concentrations. Furthermore, *Paraglomus* and *Glomus* exhibited contrasting patterns, suggesting that the karst geochemical environment acts as an environmental filter driving community reorganization.

In contrast, within karst systems, the absence of significant differences in *Glomus* abundance between bulk soil and the rock–soil interface in both dolomite and limestone indicates that microhabitat heterogeneity is insufficient to offset the dominant influence of the carbonate-derived geochemical background [55]. Although *Glomus* exhibits strong adaptive capacity across a wide range of environmental conditions, the consistently high exchangeable Ca^2+^ and alkaline geochemical background of karst soils likely provides a uniformly favorable environment, resulting in comparable relative abundances across bulk soil and the rock–soil interface [32,56]. The absence of significant differences in AMF community composition between bulk soil and the rock–soil interface contrasts with findings from other ecosystems, in which rhizosphere microhabitats often harbor distinct microbial communities [14]. This discrepancy emphasizes that lithology plays a more dominant role than soil compartment.

### 4.3. The Effect of Lithology Between Bulk Soil and Rock–Soil Interface on Co-Occurrence Network Patterns Among AMF Taxa

Previous studies have demonstrated that greater co-occurrence network complexity is closely associated with enhanced community stability [57,58,59,60]. Highly connected microbial networks tend to be more resistant to environmental stressors, such as drought and nutrient depletion [40,61]. In this study, although AMF diversity did not differ significantly among lithologies or between bulk soil and the rock–soil interface, lithology between bulk soil and the rock–soil interface significantly influenced AMF co-occurrence network properties. Although karst soils supported higher numbers of nodes and edges, their co-occurrence networks among AMF taxa exhibited lower density and transitivity, indicating a more loosely connected structure. This pattern is likely attributable to the pronounced environmental heterogeneity and strong niche partitioning in karst landscapes, which facilitate the coexistence of diverse taxa while constraining the formation of highly clustered interspecific links [13,14]. Conversely, AMF networks in non-karst soils displayed higher density, transitivity, and betweenness centrality, indicative of a more condensed and centralized topology [14]. These network features are likely driven by more homogeneous bulk soil and the rock–soil interface, which favor the establishment of stronger and more functionally integrated associations among AMF taxa [12,62]. Compared to non-karst areas, karst areas exhibit lower plant diversity, which may promote a more centralized pattern of AMF–plant interactions and potentially limit the diversification of connections among AMF taxa [63]. Simultaneously, high-nutrient environments have been shown to suppress the complexity of AMF networks [64]. Therefore, in karst habitats, the combined effects of reduced plant diversity and elevated soil nutrient availability may jointly constrain niche differentiation and functional complementarity among AMF taxa, leading to a relatively simplified network structure with lower topological properties. Notably, recent studies in karst ecosystems have reported that increased habitat heterogeneity can reduce microbial network complexity through habitat fragmentation and amplified stochastic assembly [65]. Similarly, anthropogenic disturbances have been shown to increase the diversity of soil microbial communities while simultaneously decreasing network connectivity [66]. These findings support our interpretation that the stressful heterogeneity characteristic of karst environments, such as high exchangeable Ca^2+^ and shallow and discontinuous soils, imposes strong environmental filtering process, thereby leading to a more loosely connected AMF network.

As mentioned above, there is higher microbial biomass as well as greater exchangeable Ca^2+^ and N availability in the rock–soil interface than in the bulk soil. However, contrary to our expectations, the AMF network shows no significant difference between the rock–soil interface and the bulk soil, and the only exception is that the AMF network density and average path length in the limestone is higher at the rock–soil interface than in the bulk soil. This may be attributed to the fact that interactions among AMF taxa are primarily regulated by host plants and root-associated processes [14,62]. Extensive root penetration and mycelial connectivity enable resource sharing between the rock–soil interface and bulk soil, thereby buffering the effects of localized nutrient heterogeneity [49,62]. Moreover, long-term Ca-rich conditions in karst systems likely enhance functional redundancy and network robustness in AMF communities, limiting structural reorganization even in relatively resource-enriched microhabitats [67].

Across microbial networks representing karst dolomite, karst limestone, and non-karst clastic rock soils, the majority of node and edge connections consistently involved *Glomus*, *Paraglomus*, and *Racocetra*. This recurring association underscores their role as keystone taxa in karst ecosystems, where they occupy central positions within the microbial network and contribute functionally to sustaining ecosystem stability and resilience. These findings align with previous studies in similar contexts [44,68,69].

## 5. Conclusions

In this study, we compared AMF communities between karst (dolomite and limestone) and non-karst (clastic rock) lithologies across two soil compartments (bulk soil and rock–soil interface) (Figure 6). Our results revealed that lithology exerts a stronger influence on AMF communities than soil compartment. The AMF community composition differed significantly between karst and non-karst soils, indicating that certain AMF genera exhibit distinct adaptive capacities across contrasting soil environments. Although the AMF co-occurrence network in karst soils showed a higher number of nodes and edges, its network density, transitivity, betweenness centrality, and average path length were lower than those observed in non-karst soils. Within the same lithology (dolomite and limestone), although nutrient availability was significantly higher at the rock–soil interface than in bulk soil, AMF community composition and network topological properties were highly similar between bulk soil and rock–soil interface. These findings suggest that AMF-based restoration efforts should prioritize lithological context. Moreover, plant–AMF combinations should explicitly consider the tolerance of fungal symbionts to Ca-rich and alkaline conditions, as different AMF groups exhibit contrasting responses to these karst-specific stressors.

## Data Availability

The data used to support the findings of this study can be made available by the corresponding author upon request.

## Supplementary Materials

The following supporting information can be downloaded at: https://www.mdpi.com/article/10.3390/microorganisms14051023/s1. Figure S1: Soil physicochemical properties across bulk soil and rock–soil, within both karst and non-karst forest ecosystems.
