# Supplementary material for: Lithology Controls on Arbuscular Mycorrhizal Fungi Across Bulk Soil and Rock–Soil Interface
Source: Microorganisms. 2026 Apr 30;14(5):1023. doi: 10.3390/microorganisms14051023 (PMC13209840; doi:10.3390/microorganisms14051023)
Supplement: Supplementary file 1 [file microorganisms-14-01023-s001.zip › microorganisms-4241106-supplementary.pdf]

Additional file for:

Lithology controls on arbuscular mycorrhizal fungi across bulk soil and rock-soil interfaces

Rui Pan[1,2,3†], Hao Hu[1†], Kaixun Yang[1,2,3], Dan Xiao [2,3*], Cong Wang [1*], Hanqing Wu[2,3], Qiumei Ling[2,3,4], Mingming Sun[2,3,4], Wei Zhang[2,3], Kelin Wang[2,3]

[1] Guangxi Key Laboratory of Forest Ecology and Conservation, State Key Laboratory for Conservation and Utilization of Subtropical Agro-Bioresources, School of Forestry, Guangxi University, Nanning 530004, China

[2] Institute of Subtropical Agriculture, Chinese Academy of Sciences, Changsha 410125, China

[3] Huanjiang Observation and Research Station for Karst Ecosystem, Guangxi Key Laboratory of Karst Ecological Processes and Services, Institute of Subtropical Agriculture, Chinese Academy of Sciences, Huanjiang, 547100

[4] University of Chinese Academy of Sciences, Beijing 100039, China

* Correspondence: danxiao@isa.ac.cn (D.X.); wangcuriel@foxmail.com (C.W.);

Tel: +86-0731-84619720 (D.X.); +86-17665011689(C.W.);

Fax: +86-0731-84612685 (D.X.); +86-0771-3271418(C.W.);

[†] These authors contributed equally to this work.

[*]Corresponding authors:

Dan Xiao (E-mail: danxiao@isa.ac.cn; Tel: 86-0731-84619720); **Cong Wang** (E-mail: wangcuriel@foxmail.com; Tel: 86-17665011689)

List of additional Figures

Fig. S1 Soil physicochemical properties under different lithologies and bulk soil, rock-soil interfaces in karst and non-karst areas, Southwest China.

SWC, soil water content; SOC, soil organic carbon; STN, soil total nitrogen; NH_4^+, ammonium; NO_3^-, nitrate; DON, dissolved organic nitrogen; MBN, microbial biomass nitrogen; MBC, microbial biomass carbon; $(Ca+Mg)_{exe}$, exchangeable Ca^{2+} and Mg^{2+}; $(Fe+Al)_o$, poorly crystalline oxyhydroxides of iron and aluminum; $(Fe+Al)_d$, pedogenic iron and aluminum; Clay+Silt, the sum of soil clay and silt content. DS, dolomite bulk soil; DI, dolomite rock-soil interface; LS, limestone bulk soil; LI, limestone rock-soil interface. CS, clastic rocks bulk soil. Different lowercase letters indicate significant differences among treatments ($p < 0.05$).

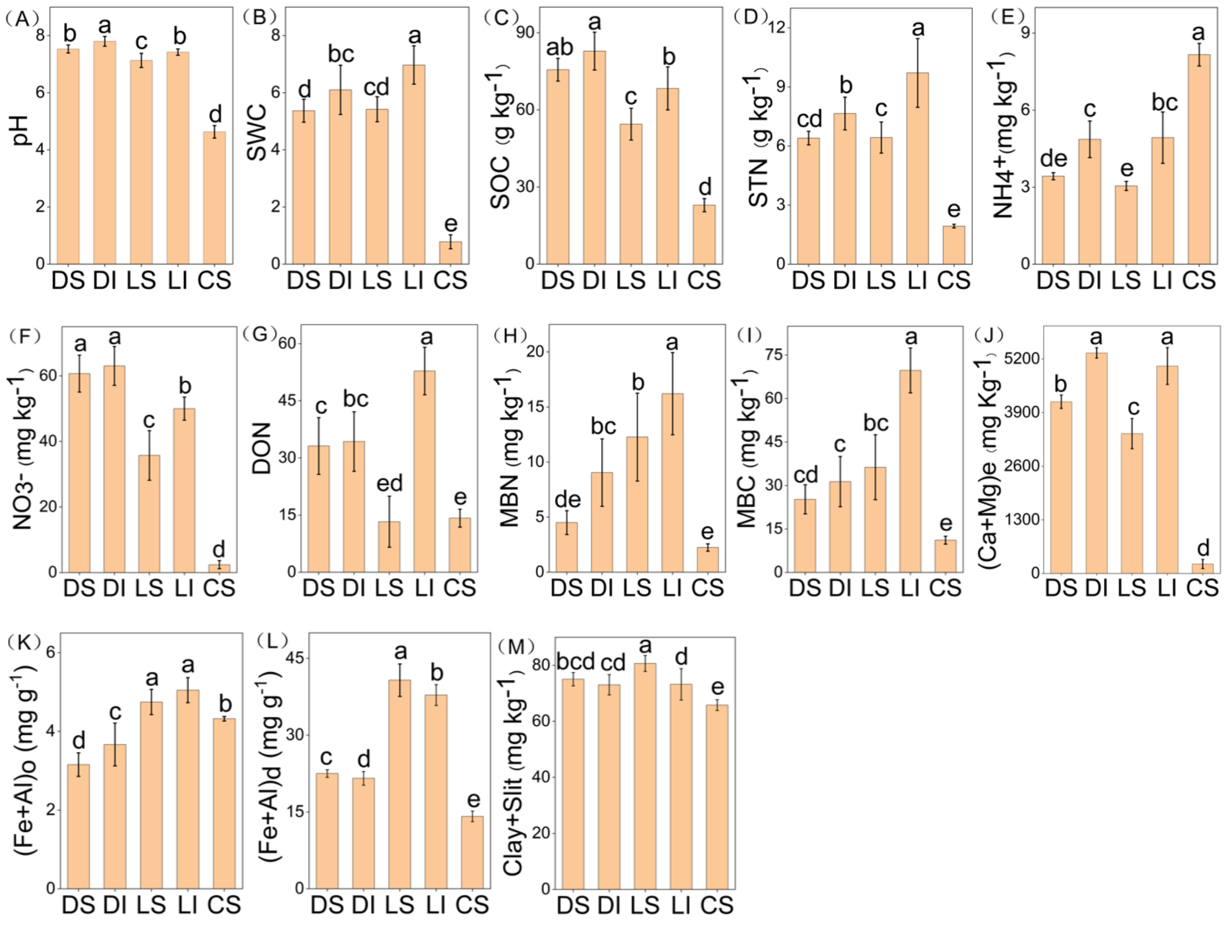

Fig. S1 Soil physicochemical properties across bulk soil, rock-soil, within both karst and non-karst forest ecosystems.

SWC, soil water content; SOC, soil organic carbon; STN, soil total nitrogen; NH_4^+, ammonium; NO_3^-, nitrate; DON, dissolved organic nitrogen; MBN, microbial biomass nitrogen; MBC, microbial biomass carbon; $(Ca+Mg)_{exe}$, exchangeable Ca^{2+} and Mg^{2+}; $(Fe+Al)_o$, poorly crystalline oxyhydroxides of iron and aluminum; $(Fe+Al)_d$, pedogenic iron and aluminum; Clay+Silt, the sum of soil clay and silt content. DS, dolomite bulk soil; DI, dolomite rock-soil interface; LS, limestone bulk soil; LI, limestone rock-soil interface. CS, clastic rocks bulk soil. Different lowercase letters indicate significant differences among treatments ($p < 0.05$).

Note, soil physicochemical properties used in this study was cited from our previous study by Ling et al., (2025).

Ling, Q.; Wu, H.; Huang, Q.; Zhao, Y.; Xie, L.; Zhang, Q.; Wan, W.; Tang, T.; Hu, P.; Xiao, D. Bedrock geochemistry regulates glomalin-related soil protein accrual in subtropical karst forest soils, Southwest China. *Ecological Indicators* **2025**, *176*, 113680.
